# Impact of 12-Month mHealth Home Telemonitoring on Clinical Outcomes in Older Individuals With Hypertension and Type 2 Diabetes: Multicenter Randomized Controlled Trial

**DOI:** 10.2196/59733

**Published:** 2025-05-29

**Authors:** Matic Mihevc, Majda Mori Lukančič, Črt Zavrnik, Tina Virtič Potočnik, Nina Ružić Gorenjec, Marija Petek Šter, Zalika Klemenc-Ketiš, Antonija Poplas Susič

**Affiliations:** 1 Department of Family Medicine, Medical Faculty University of Ljubljana, Ljubljana, Slovenia; 2 Primary Healthcare Research and Development Institute, Community Health Centre Ljubljana, Metelkova ulica 9, Ljubljana, 1000, Slovenia, 386 40520247; 3 Department of Family Medicine, Medical Faculty University of Maribor, Maribor, Slovenia; 4 Institute for Biostatistics and Medical Informatics, Medical Faculty University of Ljubljana, Ljubljana, Slovenia

**Keywords:** mobile health, aged, HbA_1c_, blood pressure, primary health care, multimorbidity, sustainability, health education, patient-centered care

## Abstract

**Background:**

As the population ages, the prevalence of chronic diseases such as arterial hypertension (AH) and type 2 diabetes (T2D) is increasing, posing challenges for effective management in primary care settings. Although mobile health (mHealth) home telemonitoring offers promising support, evidence regarding its clinical impact on older patients is limited.

**Objective:**

The objective of this paper was to evaluate the impact of 12-month telemonitoring on clinical outcomes in older individuals with AH and T2D compared to standard care in a primary care setting.

**Methods:**

In a multicenter, open-label, randomized controlled trial, individuals aged 65 years and older with AH and T2D were randomly assigned in a 1:1 ratio to either a telemonitoring group or a standard care group. The telemonitoring group received mHealth support in addition to standard care. Over 12 months, participants measured blood pressure (BP) twice weekly with 2 consecutive readings each morning and evening, using the second reading as valid. Blood glucose (BG) was measured monthly, both fasting and 90 minutes after meals. Abnormal results triggered a 7-day BP or 1-day BG profile or a teleconsultation with a general practitioner. Meanwhile, the control group received routine care based on integrated care protocols at community health centers. Primary outcomes were the differences between groups in the change in systolic blood pressure (SBP) and HbA_1c_ levels at 12 months after inclusion from baseline. Secondary outcomes included changes in diastolic blood pressure, fasting BG, lipid profile, body mass index, appraisal of diabetes, and behavioral risk factors.

**Results:**

Initially, 128 patients were enrolled, with 117 (91.4%) completing the 12-month follow-up. The mean age was 71.3 (SD 4.7) years, with a mean SBP of 136.7 (SD 14.1) mmHg and mean HbA_1c_ of 7.2% (SD 1.0%). There were no significant sociodemographic or clinical differences between groups at baseline. At 12 months, the telemonitoring group experienced significant reductions in SBP (−9.7 mmHg, 95% CI −12.6 to −6.8; *P*<.001) and HbA_1c_ (−0.5%, 95% CI −0.8 to −0.3; *P*<.001), whereas the control group exhibited nonsignificant changes in SBP (−2.8 mmHg, 95% CI −5.9 to 0.2; *P*=.07) and HbA_1c_ (0%, 95% CI −0.3 to 1.9; *P*=.75). The difference between groups at 12 months was significant for both SBP (−6.9 mmHg, 95% CI −11 to −2.7; *P*=.001) and HbA_1c_ (−0.5%, 95% CI −0.8 to −0.2; *P*=.002), with no significant differences observed in secondary outcomes.

**Conclusions:**

Telemonitoring effectively improves AH and T2D control in older people but has no impact on other cardiovascular risk factors and diabetes-related quality of life. Future research should explore combining educational and behavioral interventions with telemonitoring to enhance overall health outcomes. However, complex interventions may pose challenges for the elderly, suggesting the need for careful patient selection to ensure that benefits outweigh potential burdens.

## Introduction

The growing challenges in managing chronic diseases, specifically arterial hypertension (AH) and type 2 diabetes (T2D), highlight the need for innovative solutions. Recent changes in age distribution, health care approaches, and technological integration, along with projections of increased prevalence, particularly among the elderly, underscore the importance of developing effective strategies to address these health issues [1,2].

In response to these challenges, health care providers are increasingly adopting telemonitoring as a strategic approach to optimize integrated care processes and extend patient care beyond traditional health care settings. This approach entails the use of medical devices for the real-time collection of physiological data, including metrics such as blood pressure (BP) and blood glucose (BG) [3-6].

When coupled with teleconsultations, telemonitoring facilitates seamless communication, enabling timely adjustments to treatment plans. By emphasizing a proactive and collaborative paradigm, this approach contributes to the development of a well-informed and engaged patient population. This, in turn, has the potential to improve adherence to treatment plans and lifestyle modifications, ultimately leading to better health outcomes [4,7,8].

Combining telemonitoring and teleconsultations for at least 12 months optimizes outcomes in controlling BP and BG. After 12 months of telemonitoring, individuals with AH experienced additional mean reductions in systolic blood pressure (SBP) ranging from −4.7 to −9.7 mmHg and mean reductions in diastolic blood pressure (DBP) ranging from −1.3 to −5.1 mmHg [9-12]. For individuals with T2D, mean reductions in HbA_1c_ levels ranged from −0.03% to −0.33% at the 12-month mark [13-20]. However, the impact on lipid profile, body mass index (BMI) and quality of life was considered clinically irrelevant [13,15-18].

A notable limitation of previous research lies in the oversight of the central role of primary care in managing chronic diseases, overseeing nearly 80% of all cases of AH and T2D [6,21]. Clinical pathways, typically developed for larger clinical settings, have often overlooked the potential impact on smaller health centers. In addition, research studies have primarily focused on younger, motivated individuals dealing with isolated AH or T2D, neglecting older multimorbid patients [22].

Consequently, there is limited evidence on the clinical effectiveness of telemonitoring among the elderly in primary care. Older people face challenges such as multimorbidity, cognitive impairment, visual impairment, limited mobility, environmental dependency, and reluctance to adopt modern technologies, which may reduce its effectiveness [2,5,23,24]. Considering demographic shifts, it is imperative to evaluate the benefits of telemonitoring in this age group and to adapt interventions to address the specific needs of older people.

The objective of this study was to assess the impact of a 12-month telemonitoring intervention on clinical outcomes in older individuals with AH and T2D, comparing it with standard care in primary care settings.

## Methods

### Study Design

We conducted a multicenter, prospective, open-label, parallel, randomized controlled study as part of the SCUBY (Scale up an integrated care package for diabetes and hypertension for vulnerable people in Cambodia, Slovenia, and Belgium) project between March 2021 and June 2023. The study adhered to the published protocol [4] and was registered in the ISRCTN registry.

### Ethical Considerations

The study followed the ethical standards of the Declaration of Helsinki, with ethical approval granted by the Medical Ethics Committee of the Republic of Slovenia (0120-219/2019/4) before initiation. Informed written consent was obtained from all participants before enrollment, encompassing both primary and secondary analyses. Participants were informed of their right to withdraw from the study at any time without consequence. To safeguard privacy and confidentiality, all data were fully anonymized, with each participant assigned a unique identification code upon enrollment. No compensation was offered for participation.

### Study Setting

The research was conducted in 3 primary health care centers (PHCs) in Slovenia, each representing a different development context. PHC Ljubljana, serving a population of approximately 300,000 residents, presented an urban profile with a Gross Domestic Product (GDP) of 108% of the EU average in 2021. In contrast, PHC Trebnje and PHC Slovenj Gradec, serving approximately 50,000 residents in Eastern Slovenia, represented a rural setting with a GDP of 74% of the EU average in 2021 [25].

### Study Population, Sampling Strategy, and Randomization

The study enrolled participants aged 65 years and older with both AH and T2D. Convenient sampling was used, with patients invited by their general practitioners (GPs) during regular checkups. Upon agreement, participants were randomly assigned in a 1:1 ratio to either the telemonitoring or standard care group. Randomization was performed by a third party independent of the study.

The inclusion criteria were (1) ≥65 years of age, (2) confirmed diagnosis of AH with a 7-day mean home BP values ≥135/85 mmHg, (3) confirmed diagnosis of T2D with fasting BG value ≥7 mmol/l or venous plasma glucose ≥11.1 mmol/l 2 hours after glucose tolerance test with 75 g glucose intake or any random opportunity, (4) diagnosis of AH and T2D for at least a year, and (5) ability to use telemonitoring equipment.

The exclusion criteria were (1) <65 years of age, (2) T2D requiring insulin treatment at inclusion, (3) gestational diabetes or type 1 diabetes, (4) dementia, and (5) inability to use telemonitoring equipment for various reasons.

### Standard Care

In Slovenia, a team comprising a GP and a registered nurse delivers integrated care for patients with AH and T2D at the primary care level. The care follows established chronic integrated care protocols. Annual check-ups encompass physical examinations, laboratory tests, disease assessments, and treatment adjustments overseen by the GP. The nurse conducts screenings for complications, provides education on nonpharmacological measures, and refers patients to health promotion activities within health education centers staffed by nurses, nutritionists, psychologists, and kinesiologists. At least once a year, patients are also screened for diabetic retinopathy at retinopathy screening centers [4,26,27].

### Telemonitoring Intervention

We supported standard care by introducing a telemonitoring intervention, detailed in another paper [4]. At enrollment, each patient received an hour of training from a nurse on the measurement protocol and how to use the telemonitoring package. The package included an LG K22 smartphone and monitors for BP (A&D 651 BLE) and BG (Contour Next One). Data from these sensors were transmitted to a mobile app through Bluetooth technology, acting as a central hub. The application then transferred results to the telemedicine cloud platform using a 4G or 5G mobile standard. All received data were encrypted and adhered to the General Data Protection Regulation (GDPR).

The telemedicine center was coordinated by a GP who reviewed the recorded values and acted as a link between included patients and their GPs. Patients were managed according to recent guidelines [1,28], aiming for home BP <135/85 mmHg, fasting BG <7 mmol/l, and postprandial BG <10 mmol/l, with treatment goals adjusted for the elderly.

Over the 12-month period, participants measured their BP twice a week and BG once a month. For BP, they took readings in the morning and evening, after resting for 5 minutes while seated with back and arm supported. A total of 2 readings were taken with a programmed 1-minute interval between them, with the second considered valid. For BG, they took two readings: one in the morning on an empty stomach and another 90 minutes after lunch.

**Figure 1.**
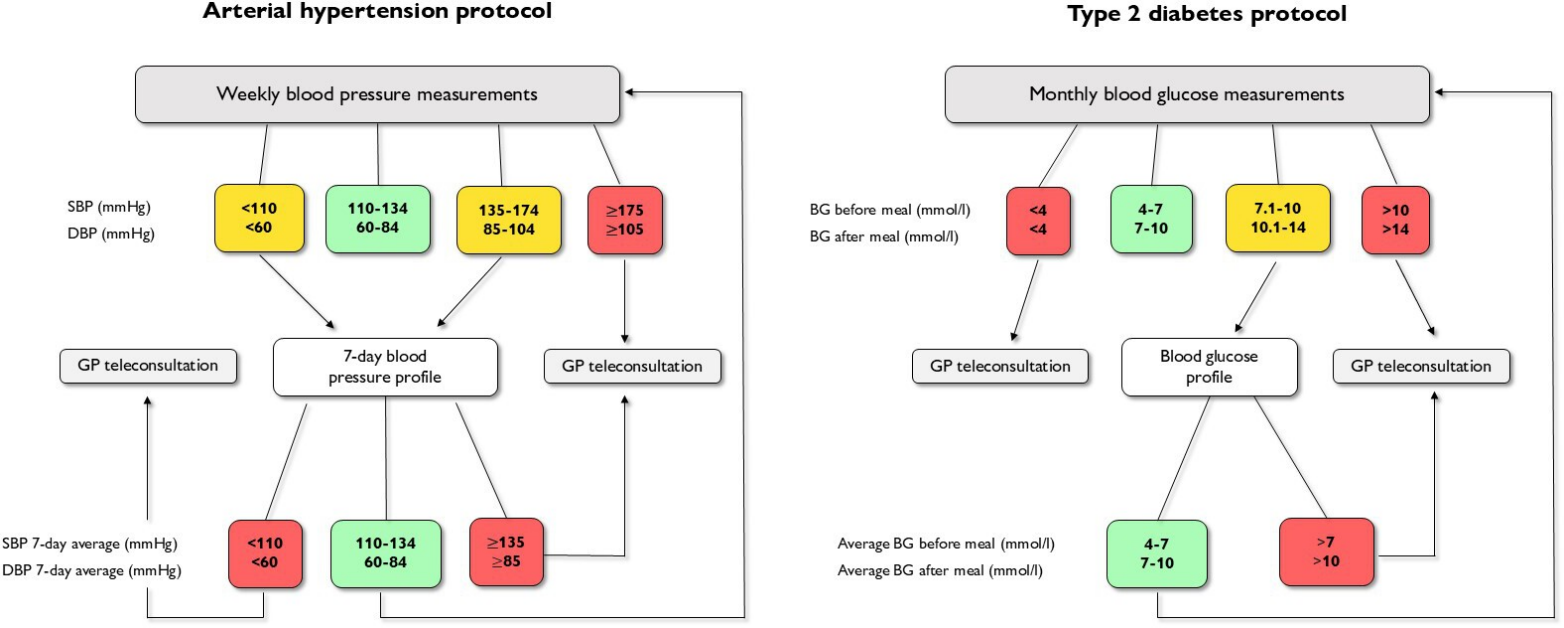
Mobile health care pathways for managing arterial hypertension and type 2 diabetes. BG: blood glucose; DBP: diastolic blood pressure; GP: general practitioner; SBP: systolic blood pressure.

The transmitted data were color-coded to indicate their status (see Figure 1). Green signaled normal values with no need to change the monitoring protocol. Yellow indicated abnormal values, triggering more intensive monitoring to better assess participants’ health. This included a 7-day BP profile with morning and evening readings for AH or a 1-day BG profile with 6 readings for T2D (values on an empty stomach, 90 minutes after breakfast, before lunch, 90 minutes after lunch, before dinner, and 90 minutes after dinner). Red indicated critically abnormal readings that required immediate teleconsultation with a GP, further evaluation, or a change in treatment plan.

### Data Collection

#### Demographic and Behavioral Profile

Participants completed a structured questionnaire providing information on demographic and behavioral characteristics, including age, gender, education, marital status, smoking, daily activity level, salt intake habits, daily number of meals, daily vegetable consumption, and alcohol consumption.

For our study, we considered physical activity adequate if it exceeded 150 minutes of moderate-intensity or 75 minutes of high-intensity per week, aligning with the World Health Organization (WHO) recommendations [4].

For alcohol consumption, we used the Slovenian version of the Alcohol Use Disorders Identification Test (AUDIT-C) to identify hazardous drinking habits. Scores over 6 points for men and 5 points for women indicated hazardous drinking habits [4].

#### Clinical History

Clinical history data included duration of AH and T2D, current treatment regimen, and accompanying medical conditions. Data were initially collected from patients and later verified in medical records.

Chronic kidney disease was defined according to the Kidney Disease Improving Global Outcomes (KDIGO) criteria as abnormalities in kidney structure (ie, albuminuria, urine sediment abnormalities, structural abnormalities detected by imaging, and history of kidney transplantation) or function (glomerular filtration rate [GFR] <60 ml/min/1.73 m^2^) that persist for at least 3 months and have significant implications for health [29].

Obesity was defined according to the WHO criteria as BMI ≥30 kg/m^2^ [30].

#### Systolic and Diastolic Blood Pressure

SBP and DBP values were measured at baseline, 6 months, and 12 months using validated monitors (A&D 651 BLE). Patients took BP readings every morning and evening, with two readings 1 minute apart. The second morning and evening reading was averaged over 7 days to establish the reference value. For telemonitored patients, data were automatically transmitted to the telemonitoring platform, while the control group manually recorded their readings. If data were missing, patients were contacted to repeat the measurements.

#### Glycemic Control, Lipid Profile, and Glomerular Filtration Rate

HbA_1c_ level, fasting BG value, lipid profile, and glomerular filtrate rate values were determined from peripheral venous blood sampling at baseline and after 12 months. In addition, fasting BG value was checked after 6 months using a validated finger-stick BG monitor (Contour Next One).

#### Body Mass Index

Body mass index was assessed at baseline and after 12 months with the measurement of body weight and height.

#### Quality of Life

Diabetes-related quality of life was measured using the Slovenian Appraisal of Diabetes Scale (ADS-S) at baseline and after 12 months. This 7-item questionnaire assesses distress, diabetes control, uncertainty, coping, and life interference, with lower scores indicating a more positive appraisal strategy [31].

### Outcomes

The primary outcomes were the differences between groups in the change in SBP and HbA_1c_ levels at 12 months after inclusion from baseline.

Secondary outcomes were differences between groups in a change in DBP, fasting BG, lipid profile, GFR, BMI, and ADS score up to 12 months after inclusion from baseline. In addition, we explored changes in selected behavioral risk factors at 12 months after inclusion from baseline (exploratory analysis).

### Sample Size and Power of the Research

Before the study began, we set a total sample size of 120 participants, with 60 in the intervention group, due to limited telemonitoring equipment. Consequently, rather than calculating the sample size required to demonstrate a clinically significant reduction (10 mmHg in SBP and 0.5% in HbA_1c_), we determined the minimum detectable difference between groups based on this planned sample size [4].

With this sample size, the study had 80% statistical power at a 0.05 significance level to detect differences in SBP changes between the telemonitoring and standard care groups at 12 months from baseline using an independent samples *t* test when the population mean difference between groups was at least 6.2 mmHg, assuming an SD of 12 in both groups, or 10.8 mmHg with a SD of 21 [10,17,32]. For changes in HbA_1c_ at 12 months from baseline, this was true when the population mean difference between groups was at least 0.7% (assuming an SD of 1.3) or 1.2% (assuming an SD of 2.4) [14,18,19,33].

### Statistical Analysis

Initially, sample distribution characteristics were assessed using the Shapiro-Wilks test. Numerical variables were summarized with mean (SD), or with median (IQR) in case of an asymmetric distribution. Categorical variables were summarized with frequencies and percentages. Between-group differences were explored using the independent samples *t* test for numerical variables with a normal distribution and the Mann-Whitney *U*-test for variables with a non-normal distribution. For categorical variables, the *χ*^2^ test was used. Within-group differences were explored using the paired-samples *t* test for numerical variables and McNemar’s test for categorical variables. We used the Holm method to adjust *P* values for multiple comparisons when reporting secondary outcomes.

Furthermore, we used mixed ANOVA analysis to examine the influence of 2 key factors—time and group—on the outcomes measured in 3 time points, namely SBP, DBP, and fasting BG. This allowed us to explore how these variables change over time within the same subjects (time factor) and how these changes differ between telemonitoring and standard care groups (group factor) within one model.

An (adjusted) *P*<.05 was considered as statistically significant. All analyses were performed using IBM SPSS Statistics for Windows (version 25.0).

## Results

### Randomization

**Figure 2.**
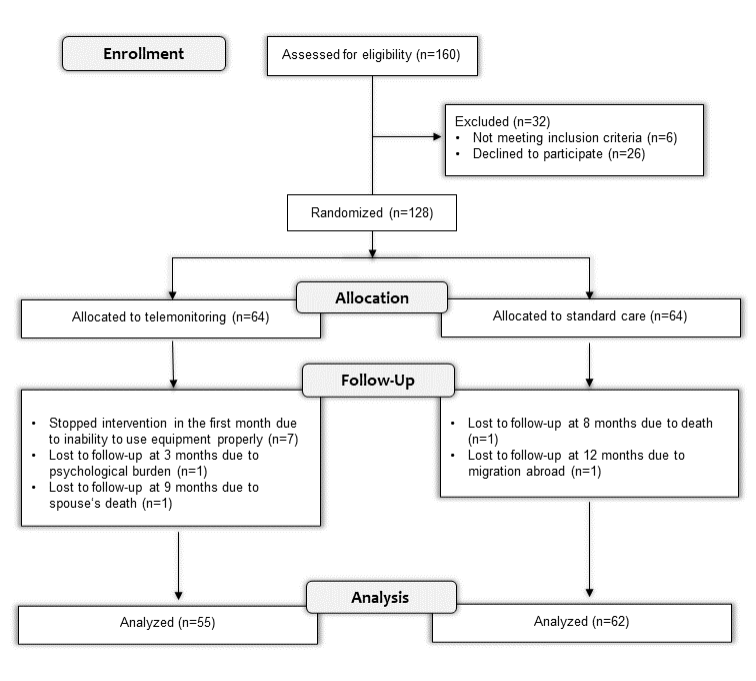
CONSORT (Consolidated Standards of Reporting Trials) flow diagram showing randomization process.

Figure 2 shows the randomization process. Among the 128 patients assigned to either the telemonitoring or standard care groups, 93.8% (120) attended the 6-month follow-up, and 91.4% (117) attended the 12-month follow-up.

### Sample Characteristics

The study included 117 participants, with a mean age of 71.3 (SD 4.7 years; range 65‐88), of whom 60.7% (71/117) were male. Most patients had completed primary or vocational school education, were married, engaged in regular physical activity, were nonsmokers, and did not report hazardous drinking habits or a tendency to add extra salt at the table. There were no significant differences between groups in terms of their sociodemographic and clinical characteristics (Table 1).

For the treatment of AH, most patients received ACE inhibitors (64/117, 54.7%), followed by calcium channel blockers (53/117, 45.3%), diuretics (51/117, 43.6%), beta blockers (34/117, 29.1%), angiotensin II receptor blockers (23/117, 19.7%), and alpha-1 antagonists (8/117, 6.8%). Conservative measures were used in 10.3% (12/117) of cases. There were no significant differences between the groups in terms of their AH treatment (see Table 2).

For the treatment of T2D, most patients received metformin (77/117, 65.8%), followed by sulphonyl urea (31/117, 26.5%), SGLT-2 inhibitor (17/117, 14.5%), DPP-4 inhibitor (6/117, 5.1%), and GLP-1 agonist (3/117, 2.6%). Conservative measures were used in 26.5% (31/117) of cases. Patients receiving standard care were significantly more commonly treated with DPP-4 inhibitor compared to telemonitoring group, whereas there were no significant differences in terms of other treatment regimens (see Table 2).

**Table 1.** Baseline sociodemographic and clinical characteristics across groups.

Characteristic	All patients (n=117)	Telemonitoring group (n=55)	Standard care group (n=62)	*P* value
Age, years, mean (SD)	71.3 (4.7)	70.6 (4.3)	72 (5)	.12
Gender				.81
Male, n (%)	71 (60.7)	34 (61.8)	37 (59.7)	
Female, n (%)	46 (39.3)	21 (38.2)	25 (40.3)	
Education				.52
Primary care, n (%)	18 (15.4)	7 (12.7)	11 (17.7)	
Vocational school, n (%)	69 (59)	33 (60)	36 (58.1)	
High school, n (%)	16 (13.7)	10 (18.2)	6 (9.7)	
Bachelor’s degree, n (%)	10 (8.5)	3 (5.5)	7 (11.3)	
Master’s degree, n (%)	4 (3.4)	2 (3.6)	2 (3.2)	
Region				.55
Urban, n (%)	54 (46.2)	27 (49.1)	27 (43.6)	
Rural, n (%)	63 (53.8)	28 (50.9)	35 (56.4)	
Marital status				.53
Married, n (%)	86 (73.5)	41 (74.5)	45 (72.6)	
Divorced, n (%)	8 (6.8)	2 (3.6)	6 (9.7)	
Widowed, n (%)	5 (4.3)	2 (3.6)	3 (4.8)	
Single, n (%)	18 (15.4)	10 (18.2)	8 (12.9)	
Duration of T2D^a^ (years), mean (SD)	9.5 (7.6)	9.8 (6.3)	9.2 (8.6)	.67
Duration of AH^b^ (years), mean (SD)	14.5 (10.6)	13.7 (10.6)	15.2 (10.6)	.46
Systolic blood pressure (mmHg), mean (SD)	136.7 (14.1)	135.8 (14.9)	137.7 (13.1)	.47
Diastolic blood pressure (mmHg), mean (SD)	76.5 (8.2)	75.6 (7)	77.2 (9)	.28
Glycated hemoglobin (%), mean (SD)	7.2 (1)	7.2 (1.2)	7.1 (0.8)	.41
Non-HDL^c^ (mmol/l), mean (SD)	3.2 (0.9)	3.1 (0.9)	3.3 (0.9)	.31
Body mass index (kg/m^2^), mean (SD)	30.4 (4.8)	30.2 (4.6)	30.5 (5.1)	.70
Glomerular filtration rate, (ml/min/1.73 m^2^), mean (SD)	77.9 (14)	80 (13.8)	76 (14.1)	.13
Chronic kidney disease, n (%)	24 (20.5)	9 (16.4)	15 (24.2)	.30
Obesity, n (%)	56 (47.9)	26 (47.3)	30 (48.4)	.90
Regular physical activity, n (%)	84 (71.8)	42 (76.3)	42 (67.7)	.30
Current smoker, n (%)	14 (12)	9 (16.3)	5 (8)	.17
Hazardous drinking habits, n (%)	16 (13.7)	10 (18.2)	6 (9.7)	.18
Salting at the table, n (%)	12 (10.3)	7 (12.7)	5 (8.1)	.39
Appraisal of diabetes score, mean (SD)	15.8 (3.6)	15.8 (3.3)	15.9 (3.4)	.84

a T2D: type 2 diabetes.

b AH: arterial hypertension.

c Non-HDL: non–high-density lipoprotein.

**Table 2.** Baseline treatment regimens across groups.

	All patients (n=117)	Telemonitoring group (n=55)	Standard care group (n=62)	*P* value
Treatment of arterial hypertension, n (%)				
ACE^a^ inhibitor	64 (54.7)	28 (50.9)	36 (58.1)	.44
Angiotensin II receptor blocker	23 (19.7)	12 (21.8)	11 (17.7)	.58
Diuretic	51 (43.6)	21 (38.2)	30 (48.4)	.27
Calcium channel blocker	53 (45.3)	23 (41.8)	30 (48.4)	.48
Beta blocker	34 (29.1)	14 (25.5)	20 (32.3)	.42
Alpha-1 antagonist	8 (6.8)	5 (9.1)	3 (4.8)	.36
Conservative	12 (10.3)	8 (14.5)	4 (6.5)	.15
Number of antihypertensive medication classes, median (IQR)	2 (1-3)	2 (1-3)	2 (1-3)	.32
Treatment of type 2 diabetes, n (%)				
Metformin	77 (65.8)	36 (65.5)	41 (66.1)	.94
Sulphonyl urea	31 (26.5)	13 (23.6)	18 (29)	.51
GLP-1^b^ agonist	3 (2.6)	1 (1.8)	2 (3.2)	.63
SGLT-2^c^ inhibitor	17 (14.5)	8 (14.5)	9 (14.5)	>.99
DPP-4^d^ inhibitor	6 (5.1)	0 (0)	6 (9.7)	.02
Conservative	31 (26.5)	15 (27.3)	16 (25.8)	.86
Number of antidiabetic medication classes, median (IQR)	1 (0‐2)	1 (0‐2)	1 (0‐2)	.45

a ACE: angiotensin converting enzyme.

b GLP-1: glucagon-like peptide-1.

c SGLT-2: sodium-glucose transport protein 2.

d DPP-4: dipeptidyl peptidase 4.

### Primary Outcomes

The telemonitoring group demonstrated significant reductions in both SBP and HbA_1c_ levels compared to the baseline up to 12 months after enrollment (see Table 3). The mean differences between telemonitoring and control group for SBP change from baseline were –10.5 mmHg (95% CI −14.6 to −6.5; *P*<.001) at 6 months and −6.9 mmHg (95% CI −11 to −2.7; *P*=.001) at 12 months. For HbA_1c_ change from baseline, the mean intergroup difference at 12 months was −0.5% (95% CI −0.8 to −0.2; *P*=.002).

Mixed ANOVA indicated significant temporal changes in SBP across both groups (*F*_2,114_=26.93; *P*<.001; η²=0.19). In addition, a significant time-group interaction (*F*_2,114_=15.08; *P*<.01; η²=0.12) highlighted differing effects on SBP between telemonitoring and control groups.

**Table 3.** Change in primary outcomes over 12 months with effect sizes.

Group	Baseline		6 months		12 months		Mean 6-month change within groups(95% CI)	*P* value	Mean 12-month change within groups(95% CI)	*P* value	Mean 6-month change between groups(95% CI)	*P* value	Mean 12-month change between groups(95% CI)	*P* value
	n	Mean (SD)	n	Mean (SD)	n	Mean (SD)								
Systolic blood pressure (mmHg)														
Intervention	55	135.8 (14.9)	55	124.7 (11.2)	55	126.2 (10.4)	–11.2(–14.1 to –8.3)	<.001	–9.7(–12.6 to –6.8)	<.001	–10.5(–14.6 to –6.5)	<.001	–6.9(–11 to –2.7)	.001
Control	62	137.7 (13.1)	62	137.1 (9.4)	61	134.9 (9.6)	–0.7(–3.5 to 2.2)	.66	–2.8(–5.9 to 0.2)	.07		N/A^a^		N/A
Glycated hemoglobin (%)														
Intervention	55	7.2 (1.2)	N/A	N/A	55	6.7 (0.8)	N/A	N/A	–0.5(–0.8 to –0.3)	<.001	N/A	N/A	–0.5(–0.8 to –0.2)	.002
Control	62	7.1 (0.8)	N/A	N/A	55	6.7 (0.8)	N/A	N/A	0(–0.3 to 1.9)	.75		N/A		N/A

a N/A: Not assessed.

### Secondary Outcomes

The difference between groups in fasting BG change from baseline was −1 mmol/l at 6 months (95% CI −1.5 to −0.4; *P*<.001) and −0.8 mmol/l at 12 months (95% CI −1.4 to −0.2; *P*=.006). However, after adjustment for multiple comparisons, the 12-month result narrowly missed statistical significance with *P*=.06.

Although telemonitoring group demonstrated statistically significant reductions in DBP at 6 and 12 months, they were not substantial. The difference between groups in DBP change from baseline was −3.4 mmHg (95% CI −6 to −0.9; *P*=.007) at 6 months and −1.7 mmHg (95% CI −4.2 to 0.7; *P*=.15) at 12 months with adjusted *P*=.81.

In terms of other secondary outcomes, there was a significant between-group difference in low-density lipoprotein (LDL) levels after 12 months compared to baseline (0.3 mmol/l, 95% CI 0.1 to 0.6), while between-group differences in total cholesterol, high-density lipoprotein, triglycerides, BMI, ADS score, and GFR were not significant. However, after adjusting *P* values for multiple comparisons, the difference in LDL was also not significant (see Table 4).

Finally, in exploratory assessment of changes in behavioral risk factors control there were no significant changes in either group (Table 5).

**Table 4.** Change in secondary outcomes over 12 months with effect sizes.

Group	Baseline		6 months		12 months		Mean 6-month change within groups(95% CI)	*P* value	Mean 12-month change within groups(95% CI)	*P* value	Mean 6-month change between groups(95% CI)	*P* value	Mean 12-month change between groups(95% CI)	*P* value	*P* value (adjusted for 12-month change between groups^a^
	n	Mean (SD)	n	Mean (SD)	n	Mean (SD)									
Diastolic blood pressure (mmHg)															
Intervention	55	75.6 (7)	55	71.2 (6.4)	55	71.6 (5.8)	−4.4(−5.7 to −3)	<.001	−4(−5.4 to −2.6)	<.001	−3.4(−6 to −0.9)	.007	−1.7(−4.2 to 0.7)	.15	.81
Control	62	77.2 (9)	62	76.3 (8.1)	61	75 (7.6)	−0.9(−3 to 1.2)	.38	−2.3(−4.2 to −0.3)	.02		N/A^b^		N/A	
Fasting blood glucose (mmol/l)															
Intervention	55	8.3 (1.5)	55	7.3 (1.3)	55	7.5 (1.6)	−1(−1.3 to −0.7)	<.001	−0.8(−1.1 to −0.6)	<.001	−1(−1.5 to −0.4)	<.001	−0.8(−1.4 to −0.2)	.006	.06
Control	62	7.9 (1.4)	60	8 (1.9)	61	8 (2.2)	0(−0.4 to 0.4)	.91	+0.1(−0.5 to 0.6)	.76		N/A		N/A	
Total cholesterol (mmol/l)															
Intervention	55	4.5 (1)	N/A	N/A	55	4.5 (1.1)	N/A	N/A	0(−0.2 to 0.2)	.95	N/A	N/A	+0.3(−0.1 to 0.6)	.12	.81
Control	61	4.7 (1)	N/A	N/A	61	4.3 (1)	N/A	N/A	-0.4(−0.6 to −0.1)	.002		N/A		N/A	
Low-density lipoprotein (mmol/l)															
Intervention	55	2.5 (0.7)	N/A	N/A	55	2.6 (1)	N/A	N/A	+0.1(−0.1 to 0.3)	.48	N/A	N/A	+0.3(0.1 to 0.6)	.03	.21
Control	60	2.7 (0.7)	N/A	N/A	61	2.4 (0.8)	N/A	N/A	−0.3(−0.5 to −0.1)	.002		N/A		N/A	
High-density lipoprotein (mmol/l)															
Intervention	55	1.4 (0.3)	N/A	N/A	55	1.3 (0.3)	N/A	N/A	-0.1(−0.2 to −0.1)	<.001	N/A	N/A	0.0(−0.1 to 0.1)	.68	≥.99
Control	61	1.4 (0.5)	N/A	N/A	61	1.3 (0.4)	N/A	N/A	−0.1(−0.2 to −0.1)	<.001		N/A		N/A	
Triglycerides (mmol/l)															
Intervention	55	1.6 (0.7)	N/A	N/A	55	1.5 (0.7)	N/A	N/A	−0.1(−0.2 to 0.1)	.38	N/A	N/A	−0.2(−0.5 to 0.1)	.29	≥.99
Control	61	1.6 (0.8)	N/A	N/A	61	1.7 (1.3)	N/A	N/A	+0.1(−0.2 to 0.3)	.54		N/A		N/A	
Body mass index (kg/m^2^)															
Intervention	54	30.2 (4.6)	N/A	N/A	54	30.6 (4.9)	N/A	N/A	+0.3(−0.1 to 0.8)	.10	N/A	N/A	+0.5(-0.1 to 1)	.12	.81
Control	59	30.5 (5.1)	N/A	N/A	59	30.4 (4.9)	N/A	N/A	−0.1 (−0.5 to 0.3)	.56		N/A		N/A	
Glomerular filtration rate (ml/min/173 m^2^)															
Intervention	54	80 (13.8)	N/A	N/A	55	78.5 (13.5)	N/A	N/A	−1.7(−3.1 to −0.2)	.03	N/A	N/A	0(−4.8 to 4.9)	.98	≥.99
Control	61	76 (14.1)	N/A	N/A	62	74.7 (13.8)	N/A	N/A	−1.5(−3 to −0.1)	.049		N/A		N/A	
Appraisal of diabetes score															
Intervention	55	15.8 (3.3)	N/A	N/A	55	15.9 (3)	N/A	N/A	+0.1(−0.9 to 1.1)	.88	N/A	N/A	+0.2(−1.2 to 1.5)	.80	≥.99
Control	62	15.9 (6.4)	N/A	N/A	60	15.9 (3.4)	N/A	N/A	−0.1(−1 to 0.8)	.83		N/A		N/A	

a Adjusted *P* values for multiple comparisons between groups at 12 months regarding all secondary outcomes in Table 4.

b Not assessed; indicates that at this time point the secondary outcome was not assessed. We assessed it only at baseline and after 12 months as per study protocol.

**Table 5.** Change in behavioral risk factors over 12 months with effect sizes (exploratory analysis).

Group	Baseline		12 months		Relative 12-month change within groups, %	*P* value
	n^a^ (%)	n^b^	n^a^ (%)	n^b^		
Regular physical activity						
Intervention	42 (76.3)	55	36 (65.5)	55	−10.8	.11
Control	42 (67.7)	62	32 (54.2)	59	−13.5	.07
Harmful drinking habits						
Intervention	7 (13)	55	8 (14.5)	55	+1.5	≥.99
Control	5 (8.1)	62	5 (8.3)	60	+0.2	.75
Salting at the table						
Intervention	10 (18.2)	54	6 (11.1)	54	−7.1	≥.99
Control	6 (9.7)	62	4 (6.8)	59	−2.9	≥.99
Having 3‐5 meals/day						
Intervention	49 (89.1)	55	48 (87.3)	55	−1.8	≥.99
Control	59 (95.2)	62	56 (93.3)	60	−1.9	≥.99
Eating vegetables daily						
Intervention	39 (70.9)	55	37 (67.3)	55	−3.6	.83
Control	44 (71)	62	38 (63.3)	60	−7.7	.49
Smoking						
Intervention	9 (16.4)	55	11 (20)	55	+3.6	≥.99
Control	5 (8.1)	62	8 (13.3)	60	+5.2	≥.99

a Represents the number of participants at inclusion.

b Represents the number of participants remaining after 12 months.

## Discussion

### Principal Findings and Comparison With the Existing Literature

The major finding of this study is that telemonitoring has been found to be a valuable strategy for improving BP and BG control in older people with AH and T2D in primary care settings. However, there were no significant improvements in control over other cardiovascular risk factors or diabetes-related quality of life.

The difference in SBP change between groups was significant with a decrease of −10.5 mmHg (95% CI −14.6 to −6.5) after 6 months and −6.9 mmHg (95% CI −11 to −2.7) after 12 months compared to the control group. From a clinical standpoint, a 6-month SBP reduction correlates with a 20% lower risk of major cardiovascular events, whereas a 12-month SBP reduction correlates to a 10%‐15% lower risk of cardiovascular events [33]. The observed 12-month SBP difference aligns with findings from previous studies, where a similar difference at 12 months ranged from −4.7 to −9.7 mmHg in patients with AH [10,34] and from −0.2 to −8.2 mmHg in patients with AH and T2D [11,17,18].

In contrast, the difference in DBP change between groups was significant after 6 months, with a decrease of −3.4 mmHg (95% CI −6 to −0.9) but became nonsignificant after 12 months, showing a decrease of −1.7 mmHg (95% CI −4.2 to +0.7). Similar patterns were observed in previous studies, where the DBP between-group difference at 12 months ranged from −1.3 to −5.1 mmHg in patients with AH [10,34], and from −0.4 to −0.7 mmHg in patients with AH and T2D [17,18]. Clinically, both outcomes are considered irrelevant, failing to meet the 5 mmHg DBP decrease threshold associated with a 20% reduction in the risk of major cardiovascular events [35].

In terms of glycemic control, we found a significant between-group difference in HbA_1c_ change of −0.5% (95% CI −0.8 to −0.2) after 12 months. In addition, a clinically meaningful between-group difference in fasting BG of −0.8 mmol/l (95% CI −1.4 to −0.2) was found after 12 months. These findings contrast with previous studies, where HbA_1c_ reductions at 12 months were less pronounced, ranging from −0.03% to −0.32% in patients with isolated T2D [13-16,19] and from +0.14 to −0.33% in patients with both AH and T2D [11,17,18,36]. Recent meta-analysis also indicates that telemonitoring in primary care produced a smaller HbA_1c_ reduction of −0.21% (95% CI −0.37 to −0.06) in patients with AH and T2D after 12 months [37]. A 0.5% reduction in HbA_1c_ is clinically significant and is associated with an estimated 17% reduction in cardiovascular disease risk. The extent of risk reduction is, however, curvilinear and dependent on baseline HbA_1c_ status and treatment regimens [38,39].

Furthermore, there were no significant differences between groups in terms of lipid profile, BMI, and GFR after 12 months. This aligns with earlier studies in patients with T2D and/or AH, where intergroup reductions for LDL ranged from −0.16 to +0.04 mmol/l [13,15-18,36]; for triglycerides, it ranged from −0.15 to +0.07 mmol/l [13,16-19]; for BMI, it remained stable or slightly increased [15,17,18]; and for GFR, it improved up to 3 ml/min/1.73 m^2^ [40].

Ultimately, telemonitoring alone did not significantly improve diabetes-related quality of life or lead to positive lifestyle changes. This finding is important, as patients achieved significant BP and BG control without making meaningful lifestyle changes. The positive outcomes may be attributed to other factors such as improved communication, medication adherence, early detection of exacerbations, and therapy adjustments based on accurate telemonitoring data [5,8,41]. However, this finding should be interpreted with caution as lifestyle changes were self-reported by participants. In-depth interviews with 29 telemonitoring participants provided several examples of reported lifestyle changes and a better understanding of how lifestyle choices affect BG control [42]. Notably, our intervention lacked a health education cointervention. Previous studies incorporating such interventions reported significant improvements in physical activity, alcohol intake, and diet quality, while smoking cessation rates remained unchanged [42-44].

### Implications for Practice and Sustainability

#### Establishing the Gold Standard for Telemonitoring Frequency

In our study, patients monitored their BP twice weekly and BG once monthly, with more frequent checks in case of derailments, resulting in a significant reduction in SBP and HbA_1c_ levels. Nevertheless, uncertainty persists regarding the optimal telemonitoring routine. Low-intensity BP monitoring, such as once weekly, boasts high adherence but offers less significant long-term benefits [45,46], while high-intensity BP monitoring, such as 5 to 6 times weekly, demonstrates better long-term outcomes but lower adherence [46-48]. When monitoring BG levels in insulin-naive patients, 1 measurement per week was found as effective as four measurements per week to maintain HbA_1c_ [49]. In a previous feasibility study [6], we found that less frequent monitoring (ie, 7-day BP profile and one-day BG profile at baseline, 2 weeks, 3 months, 6 months) was clinically irrelevant, leading to a more intensive approach in this study. However, even in this study patients exceeded the expected number of BP and BG measurements by 46.5% and 252.8%, respectively, which resulted in total mean of 2.7 BP measurements per week and 1.2 BG measurements per week [8]. Accordingly, based on results of this study and previous studies, a regimen in which BP is measured two times weekly with morning and evening readings and BG once weekly with pre- and postprandial readings could be considered as one of the standard regimens for future research.

#### Bridging Gaps in Knowledge and Long-Term Lifestyle Modification

Our study aimed to improve BP and BG control through telemonitoring, excluding health education training, which is freely available at health education centers. Despite significant improvements in primary outcomes, there were no significant changes in behavioral risk factors, highlighting the need for a more comprehensive approach. Integrating telemonitoring with tailored health education and behavioral interventions such as group sessions, individual education, or telehealth tools such as phone counseling, text messaging, health applications, and web-based resources could increase effectiveness [50]. In addition, peer support groups may be a valuable alternative, allowing individuals to share experiences, provide mutual encouragement, and foster a community-based approach to health management [51].

#### Personalizing mHealth Interventions for Elderly and Multimorbid Individuals

Telemonitoring introduces an external monitoring system that may be perceived as burdensome by older patients and their caregivers, potentially leading to unintentional dropouts. In our study, older participants encountered technical challenges such as difficulty with touch screens, forgotten passwords, and device pairing. To enhance the acceptability for this demographic, telemonitoring devices should include larger screens, clear icons, voice commands, and alternative login options such as fingerprints or lock patterns. In addition, transition to continuous glucose monitoring systems may be beneficial for insulin-dependent patients [5,6,42].

For multimorbid patients, telemonitoring should take a holistic approach that considers the interplay between different conditions, medications, and treatments. Conducting an early patient assessment focused on health risks, technological literacy, and personal preferences can help tailor interventions and improve acceptability [5,6,23,42]. Integrating comprehensive monitoring of multiple health metrics, such as weight and activity levels, along with multidisciplinary support from professionals like nutritionists and kinesiologists, could further improve patient outcomes [52].

#### Optimizing Telemonitoring Costs for Sustainable Health Care Integration

For the effective and sustainable integration of telemonitoring in health care, optimizing key cost drivers is crucial. In our parent study, we conducted a bottom-up analysis to identify these factors and define variables for future cost-effectiveness analyses [8].

To comprehensively assess costs, we considered both health care provider and patient perspectives. For providers, this included infrastructure and operational costs, while for patients, it involved participation costs and changes in out-of-pocket costs. The telemonitoring intervention incurred an annual infrastructure cost of €489.4 and operational costs of €97.3 (95% CI 85.7 to 109) per patient. For patients, annual participation costs amounted to €215.6 (95% CI 190.9 to 241.1), with average annual out-of-pocket costs for both groups reported at €345 (95% CI 221 to 469). Notably, after 12 months, the telemonitoring group experienced significantly lower out-of-pocket costs (€132 vs €545; *P*<.001), largely due to reduced spending on food, dietary supplements, medical equipment, and specialist checkups compared to the standard care group [8]. All euro (€) amounts are based on the exchange rate at the time of the study, with €1 equivalent to approximately US $1.10.

One potential cost-reduction strategy involves shortening the monitoring period to focus on critical periods without compromising patient outcomes. In the 12-month follow-up, significant BP and BG improvements were seen within the first 6 months, with minimal changes afterward. This pattern aligns with previous pilot study findings [6]. Furthermore, patients reported monitoring fatigue due to regular measurements [6,42,53]. For these reasons, we propose a future model where telemonitoring is discontinued after 6 months and replaced by a less intensive self-monitoring protocol, with monthly nurse practitioner teleconsultations to review results and restart telemonitoring if needed due to exacerbations [6,10].

Furthermore, establishing a national platform could standardize infrastructure and reduce third-party costs. Developing a health app that patients can download on their personal phones, rather than providing them with additional devices for telemonitoring could further reduce equipment expenses while ensuring accessibility, however, this may raise privacy and security risks [54]. Integrating artificial intelligence into telemonitoring systems could improve data analysis, automate routine tasks, and decrease staffing requirements [55]. Finally, employing nurse practitioners as telemedicine coordinators offers a more cost-effective option than specialist-led models, allowing specialists to focus on more complex cases [5,6,8].

### Strengths and Limitations

The strength of this study is highlighted by its multicenter, randomized controlled design, the complexity of the outcomes observed, and the novelty of the population studied. In addition, we adapted the inclusion criteria to include patients with varying degrees of difficulty and control of their AH and T2D. This deliberate approach aimed to capture a diverse population typically managed at the primary care level, thereby increasing the transferability of our findings to real-world settings.

However, it is important to consider limitations that may affect the generalizability of our findings. These include convenience sampling, the exclusion of patients requiring insulin because they are not commonly treated in primary care settings, and the reliance on self-reported behavioral data, which may affect the reliability of lifestyle-related outcomes. The inclusion criterion requiring participants to operate telemonitoring devices may have introduced a bias toward individuals with greater technical skills. However, despite training, some patients experienced technical challenges, suggesting possible limitations in practical implementation. Next, we did not apply advanced imputation methods for missing data due to the dataset’s high quality and minimal missing values. For full transparency, we reported the actual sample size for each analysis and made the dataset freely accessible online. Finally, the study primarily focused on short-term outcomes, neglecting the importance of long-term (>12 months) considerations for future expansion.

### Conclusions

The 12-month telemonitoring intervention effectively improved BP and BG control in older people with AH and T2D. Despite this improvement, it did not lead to better management of other cardiovascular risk factors or an improvement in diabetes-related quality of life. To improve overall health outcomes, future research should explore integrating educational and behavioral components into telemonitoring. Furthermore, there is a need to investigate new models of remote care, such as combining short-term telemonitoring with long-term self-measurement routines while maintaining regular teleconsultations. However, the complexity of these interventions may be challenging for the elderly, highlighting the importance of careful patient selection to ensure that the benefits outweigh potential burdens.

## Supplementary material

10.2196/59733 Checklist 1 CONSORT eHeatlh checklist.

## Abbreviations

ACE: angiotensin converting enzyme
ADS: Appraisal of Diabetes Scale
AH: arterial hypertension
AUDIT-C: Alcohol Use Disorders Identification Test
BG: blood glucose
BP: blood pressure
CONSORT: Consolidated Standards of Reporting Trials
DBP: diastolic blood pressure
DPP-4: dipeptidyl peptidase 4
EU: European Union
GDP: gross domestic product
GDPR: General Data Protection Regulation
GFR: glomerular filtration rate
GLP-1: glucagon-like peptide-1
GP: general practitioner
KDIGO: Kidney Disease Improving Global Outcomes
LDL: low-density lipoprotein
mHealth: mobile health
Non-HDL: non-high-density lipoprotein
PHC: primary health care center
SBP: systolic blood pressure
SGLT-2: sodium-glucose transport protein 2
T2D: type 2 diabetes
WHO: World Health Organization
